# Opting to wear prismatic spectacles was associated with reduced neck pain in dental personnel: a longitudinal cohort study

**DOI:** 10.1186/s12891-016-1145-1

**Published:** 2016-08-17

**Authors:** Agneta Lindegård, Catarina Nordander, Helene Jacobsson, Inger Arvidsson

**Affiliations:** 1 The Institute of Stress Medicine, SE-413 19 Gothenburg, Sweden; 2 Division of Occupational and Environmental Medicine, Lund University, SE-221 85 Lund, Sweden; 3 Clinical Studies Sweden - Forum South, Unit for Medical Statistics and Epidemiology, SE-221 85 Lund, Sweden

## Abstract

**Background:**

The aim of this study was to investigate effects on perceived exertion, work ability self-reported neck pain and clinically diagnosed conditions in the neck, of an intervention with prismatic spectacles among dental personnel.

**Methods:**

In this cohort study a baseline questionnaire including questions about frequency of neck pain, perceived exertion during work and background information was distributed to dental personnel in municipal dental care units. In connection, personnel from 78 out of 110 dental care units underwent a clinical neck examination and rated their perceived work ability with the single-item question from the Work Ability Index. The study population consisted of 564 participants; 366 in the questionnaire group, 321 in the examination group, whereof 123 participated in both assessments. In total 371 belonged to the intervention group and received individually adjusted prismatic spectacles after the baseline assessments (inclusion based on self-selection) and 193 belonged to the reference group. At the 12-month follow-up the clinical examination was repeated and the same questionnaire with additional questions was completed. Fisher’s exact test or the Mann–Whitney *U* test was used to assess differences between the intervention group and the reference group at baseline. Differences at follow up between the two groups were assessed by means of Linear-by Linear association test for trends.

**Results:**

A significant decrease in neck pain (*p* = 0.047), clinical diagnoses in the neck (*p* = 0.025), and perceived exertion (*p* = 0.003) was observed at follow up for the intervention group compared to the reference group. Moreover, for the intervention group a significantly improved self-rated work ability (*p* = 0.040) was reported. Finally, opting to wear prismatic spectacles during dental work appeared to have a preventive effect on neck pain.

**Conclusions:**

Dental personnel opting to wear prismatic spectacles reduced their neck pain significantly more at follow up compared with the reference group. These results are worthwhile testing in a randomised design. The practical implication of this study is that recommendations regarding ergonomics for dental professionals may include the use of prismatic glasses, both as primary and secondary prevention of work-related neck pain. Such glasses should also be tested in other working situations where the work tasks include high visual demands in sustained awkward neck postures.

**Electronic supplementary material:**

The online version of this article (doi:10.1186/s12891-016-1145-1) contains supplementary material, which is available to authorized users.

## Background

Symptoms and diseases in muscles and joints are common in the general population and thus constitute a major public health problem [1–3]. A large proportion of these symptoms/disorders, regardless of localisation, may be work related [4–7]. Increased risk has been demonstrated for both physical [8, 9] and psychosocial factors [10–12]. A variety of physical exposures, constrained postures, workstation layout, and working technique, have been identified as possible risk factors [13–16]. All of these risk factors are frequently present in dentistry, particularly during work in the oral cavity [17]. Consequently, work-related neck/shoulder symptoms are very common among dentists and dental hygienists around the world [5, 18, 19]. Studies focusing on one major risk factor, namely exposure to extreme head and neck forward flexion (≥45 °), have shown that 10 % of the total working time during clinical dental work is spent in this position [20]. The relationship between exposure to excessive forward bending/flexion of the neck/head (>20 °), and the risk of developing neck pain has also been investigated in previous studies, showing a doubled risk (RR = 2.0) of neck pain when working with a forward neck flexion greater than 20 ° for more than 70 % of the working time [21]. The duration of a hazardous exposure is also of importance for the risk of developing neck pain at work [20, 22]. Neck posture during dental work in the oral cavity is primarily governed by requirements on sight and precision, and efforts have previously been made to improve equipment ergonomically in order to obtain a more upright position of the neck [23, 24]. However, clinical dental work in the oral cavity still requires a rather pronounced forward flexion of the neck to satisfy the visual demands of the work task [25–27].

The significance of non-optimal viewing conditions in the occurrence of symptoms in the neck/shoulders has been demonstrated in several studies [28–31], as has the impact of various kinds of lenses in conjunction with visually demanding work [24, 31–33]. Eyeglasses with prismatic lenses have been available on the market since 2007. These lenses are similar to bifocal lenses, but the lower part of the lens is replaced by prisms that bend the light path downwards; currently by 5 °. A small pilot study (*n* = 15) on dentists with chronic neck pain (pain duration longer than 6 month) in the Netherlands, although not internationally published, has indicated that the use of prismatic spectacles led to reduced discomfort and a decrease in self-reported pain in the neck and shoulders six months after the start of the intervention. However, since this study lacked a reference group, the results could only be interpreted with caution. In order to explore the potential effects of the prismatic spectacles on the exposure to forward neck bending in a more appropriate way, a randomized controlled study was carried out comprising 45 dentists/dental hygienists in different municipal dental care units in the Region of Västra Götaland in Sweden, serving 1.5 million inhabitants in Gothenburg and the surrounding areas. The results showed that prismatic spectacles reduced exposure to extreme forward bending of the head/neck during clinical dental work [34], leading to the conclusion that the use of prismatic spectacles may reduce the risk of work-related neck pain/disorders in dental personal. The above mentioned study led to the decision by this health care region in 2010 to allocate funds for the general implementation of prismatic spectacles in all dental care units in the region. This enabled our research team to follow the effects of this large-scale intervention in close co-operation with the human resources department in the region of Västra Götaland. Hence, the main aim of this study was to investigate the effects on self-reported neck pain, clinically diagnosed conditions in the neck, perceived exertion, and self-reported work ability among dental personal opting to use prismatic glasses during clinical dental work.

## Study design and procedure

In order to involve all stakeholders in the intervention, an operational project group was formed. This group consisted of one researcher (the first author of this paper), one ergonomist, the head of the Human Resources Department for all the dental clinics, and a coordinator from the same human resources department, all of whom were employed by the Västra Götaland Region, apart from the researcher. Finally, a representative from the company supplying the prismatic spectacles was also included in the project group. This group was responsible for the implementation of the intervention. Another group consisting of researchers (all authors of this paper) was formed to organize and perform the scientific evaluation of the intervention.

To evaluate the effects of the intervention, the group given prismatic spectacles was compared with a reference group not given prismatic spectacles in a longitudinal study design. Both questionnaires and clinical examinations were used in order to improve the reliability of the outcome measurements, and thus attain results with high credibility [35]. In the intervention group all measurements, including the clinical examination, were performed at baseline and 12 months after the intervention group was given the glasses. Those belonging to the reference group were examined clinically by the ergonomists twelve months after the baseline examination was performed.

### Study population

**Fig. 1:**
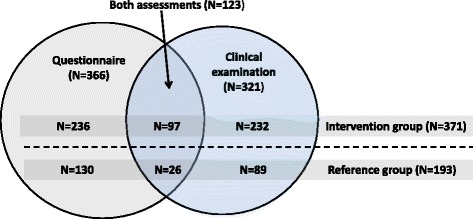
Distribution of the study population (N=564), of participants in the questionnaire survay (N=366), the clinical examination (N=321) and both assessments (N=123), stratified by intervention group (prismatic glasses; N=371) and reference group (N=193)

All dental clinics in the region were contacted by the human resources department, mutual for all the clinics. A coordinator in each clinic was responsible for spreading the information about the project to all eligible personnel (*n* = 1184). The coordinators were also responsible for receiving the declarations of interest from potential participants and for sending them forward to the human recourses department. The total amount of money reserved for the implementation was enough to support about 50 % of all the dentists, dental hygienists and orthodontic assistants with prismatic glasses. Each clinic could decide whether to participate or not. Altogether, 78 out of 110 dental care units (71 %) within the region were represented in the study. The final study population included those 564 individuals (men *n* = 121, women *n* = 443) who had reported interest in receiving the prismatic glasses and had answered the questionnaire, both at baseline and at follow-up, or had attended the clinical examination both at baseline and at follow-up (or both of these; Fig 1). Three different dental professions were represented, dentists (*n* = 355), dental hygienists (*n* = 173) and orthodontic assistants (*n* = 36). The proportion included from each profession represented these three categories employed by the dental units in the Västra Götaland Region. Since each dental unit was allowed to decide whether they would participate in the intervention or not, no randomization took place. Further, all dentists, dental hygienists, and orthodontic assistants who wanted to try the prismatic glasses were offered them. The remaining subjects with these occupations formed the reference group. The intervention group consisted of 371 individuals and the reference group comprised 193 individuals. Baseline data are given in Table 1, for the two groups separately.

**Table 1:** Baseline characteristics of the subjects given prismatic glasses and the reference group. Values presented are number (%) or median (min-max)

	Intervention group	Reference group	p-value^a^
	*N* = 371	*N* = 193	
Women, n (%)	292 (79)	151 (78)	0.914
Age (years), n (%)	50 (23–66)	51 (23–73)	0.078
Profession, median (range)			
Dentists	243 (65)	112 (58)	
Hygienists	105 (28)	68 (35)	
Orthodontic assistants	23 (6)	13 (7)	0.195
Questionnaire	*n* = 236	*n* = 130	
Dental work (hours/week), n (%)			
0–29	75 (32)	49 (38)	
30–40	161 (68)	81 (62)	0.299
Perceived exertion^b^ n (%)			
0–7	124 (53)	72 (58)	
>7	110 (47)	53 (42)	0.437
Complaints frequency^c^ n (%)			
2 times a month or less	83 (35)	58 (45)	
Once a week or more	153 (65)	70 (55)	0.071
Clinical examination (HECO)	*n* = 232	*n* = 89	
Diagnoses in neck/shoulders, n (%)	39 (17)	6 (7)	0.020
Neck/shoulder pain^d^ n (%)	129 (56)	36 (41)	0.024
Work ability^e^ median (range)	9 (3–10)	9 (4–10)	0.588

^a^Fisher’s exact test or the Mann–Whitney *U* test

^b^2 subjects missing in the intervention group and 5 subjects missing in the reference group

^c^2 subjects missing in the reference group

^d^1 subject missing in the reference group

^e^22 subjects missing in the intervention group and 11 missing in the reference group

## Methods

### Baseline questionnaire

A baseline postal questionnaire comprising questions about age, sex, profession, hours of clinical work per week, frequency of complaints in the neck/shoulders during the preceding 12 months, perceived exertion in the neck/shoulders after a typical working day, perceived comfort at work, was sent out to all dentists, dental hygienists and orthodontic assistants (*n* = 1184) employed by the Region of Västra Götaland. The questionnaire was returned by 538 individuals (344 dentists, 153 dental hygienists and 38 orthodontic assistants).

### Frequency of complaints

The participants were asked about subjective musculoskeletal complaints from the neck and shoulder area during the past 12 months. The response alternatives were: a) never, b) 1–2 times/month, c) at least once a week, and d) almost every day. In the analysis, alternatives a and b were combined into one category (complaints 2 times per month or less = unexposed), and alternatives c and d were combined into another category (once a week or more = exposed).

### Perceived exertion

Perceived exertion during work was rated on a modified Borg perceived exertion scale ranging from 0 to 14 [36]. In the analysis, the data were dichotomized at the median value for the study population; participants scoring 0–7 were considered to have low exposure, and participants scoring >7 were regarded as having high exposure [36].

### Clinical examinations

The clinical examination at baseline was performed by 10 specially trained ergonomists from the occupational health services in the region of Västra Götaland and consisted of a short screening part and a detailed physical examination part. The detailed examination part was only performed if the screening indicated ongoing symptoms. The examinations were conducted according to the Health Surveillance in Adverse Ergonomics Conditions HECO protocol elaborated to allow clinical diagnosis of neck and shoulder musculoskeletal disorders in professions exposed to ergonomic risk factors and described more in detail in a recently published paper by Jonker and co-workers [37]. All available dentists, dental hygienists and orthodontic assistants in the participating 78 dental units were assessed. In total 452 individuals were clinically examined at baseline.

### Musculoskeletal pain

In connection with the clinical examination, the participants were asked to report subjective musculoskeletal pain during the preceding 12 months, according to the Nordic Musculoskeletal Questionnaire [38]. Information was also collected on the frequency of neck complaints during the past year on a 5-point scale (1 = never, 2 = seldom, 3 = sometimes, 4 = often or 5 = very often [39], as well as the intensity of pain on a ten-point scale, from 0 (none at all) to 10 (extremely strong) [40]. A participant was considered to have substantial musculoskeletal pain (subsequently referred to as *pain*) if pain was reported at least “seldom” with an intensity of at least 7 (very strong pain), or pain was reported “sometimes” with an intensity of at least 3 (moderate pain), or “often” or “very often” with an intensity of at least 2 (slight/mild) [41].

### Work ability

Self-rated work ability was measured according to the single-item question from the work ability index by Tuomi and Ilmarinen [42] in which the current work ability is compared with the lifetime best, with a possible score ranging from 0 (completely unable to work) to 10 (work ability at its best). This single item question has been used frequently in clinical practice and research [43, 44], and has been validated by Åhlström and co-workers [45]. The response alternatives were dichotomized such that responses ranging from 0 to 8 were combined and characterized as reduced work ability, and responses ranging from 9 to 10 were regarded as indicating good work ability.

### The intervention

An optician from the provider of the prismatic glasses, and one of the trained ergonomists from the occupational health service coordinated their visits to the dental care units. Individuals who had applied to participate in the intervention were given an eye test by the optician. Participants in the intervention group were then prescribed prismatic glasses. Optometric correction was included when necessary. All participants were given information by the ergonomist on specific working techniques during a 10-minute session (e.g. shorter distance between the patient and the professional) [34]. Prismatic glasses enable the user to work in a more upright position with a less bent neck, as described in the introduction. A photo of the prísmatic glasses are shown in Additional file 1. For more details concerning the reduction of neck flexion obtained by the prismatic spectacles we refer the reader to an earlier paper by Lindegård and co-workers [34].

### Follow-up measurements

#### Questionnaire

At the 12-month follow-up, the baseline questionnaire was repeated including additional questions. They were asked about which group they belonged to (the intervention group or the reference group), and those in the intervention group answered questions on the compliance and usability of the prismatic glasses in various working situations. Compliance was determined by asking whether they had used the glasses “daily in all kinds of dental work”. The response alternatives were either yes or no.

#### Clinical examination

The follow-up HECO examination was carried out 12 months after the participants had received their prismatic glasses, by the same ergonomists who performed the baseline examinations. The ergonomists had no knowledge of which group the participants belonged to. Among those who underwent the HECO examinations, data on compliance are only available for those who also filled in the follow-up questionnaire, since compliance was not ascertained during the HECO examination.

## Statistical analysis

Baseline characteristics are presented as number and percent, or medians with minimum and maximum values. Fisher’s exact test or the Mann–Whitney *U* test was used to assess differences between the intervention group and the reference group. Changes in frequency of complaints, perceived exertion, musculoskeletal pain, one or more diagnoses and work ability, compared with baseline, were categorized into worse, unchanged or better (based on dichotomised outcome measures). Changes in the frequency of complaints and perceived exertion were stratified by frequency of complaints (high/low) at baseline for the subjects who had been given prismatic glasses and stated that they used them on a regular basis, as well as from the controls who participated in the questionnaire/examination? (Table 3). Participants in the questionnaire group who received prismatic glasses but reported that they did not use them on a regular basis were excluded from the analysis (*n* = 66). Data from those who participated in the HECO examination (both the intervention group and the reference group) was also stratified according to neck/shoulder pain (yes/no) at baseline (Table 4). In conformity with the procedure described above, participants in the HECO examination group who received prismatic glasses but reported that they did not use them on regular basis were excluded (*n* = 45). Stratification was carried out as we assumed that individuals with pain or frequent complaints would be more prone to try the prismatic glasses in the first place, causing bias in the self-selection inclusion procedure. Furthermore, symptomatic individuals, per se, have greater potential for improvement with respect to existing symptoms.

The changes are given as number and percent. Differences between the intervention group and the reference group were evaluated by means of Linear-by-Linear association test for trends and a p-value below 0.05 indicated statistically significant difference. All statistical analyses were performed using SPSS: IBM Statistics for Windows version 22 (IBM Corporation, New York, NY, USA).

## Results

### Baseline

At baseline, the intervention group reported a higher prevalence of neck/shoulder pain (56 %, *n* = 129) and also exhibited a higher prevalence of clinical diagnoses in the neck/shoulders (17 %, *n* = 39), than the reference group (40 %, *n* = 36 and 7 %, *n* = 6). Apart from this anticipated difference (due to self-selection into the intervention group), no other major differences were found between the two groups at baseline (Table 1).

### Follow-up

When analysing the results from the whole study group (*n* = 564) we found significant improvements regarding clinical diagnoses (*p* = 0.025), perceived exertion (*p* = 0.003), self-reported pain (*p* = 0.047) and self-rated work ability (*p* = 0.040) in the intervention group compared to the reference group (Table 2).

**Table 2:** Changes between baseline and follow-up in prevalence of symptoms and diagnoses in neck and/or shoulders for the subjects who received prismatic glasses (Intervention) as well as from the referents (Referent)

	Intervention	Referent	p-value^a^
Questionnaire	*n* = 236	*n* = 130	
Complaints frequency			
Worse	21 (9)	26 (20)	
Unchanged	123 (52)	57 (44)	
Better	92 (39)	47 (36)	0.060
Perceived exertion^b^			
Worse	55 (24)	44 (35)	
Unchanged	49 (21)	33 (26)	
Better	129 (55)	48 (38)	0.003
Clinical examination (HECO)	*n* = 232	*n* = 89	
Musculoskeletal pain^c^			
Worse	20 (9)	13 (15)	
Unchanged	167 (74)	66 (75)	
Better	39 (17)	9 (10)	0.047
One or more diagnoses			
Worse	19 (8)	9 (10)	
Unchanged	186 (80)	79 (89)	
Better	27 (12)	1 (1)	0.025
Work ability			
Worse	51 (25)	23 (30)	
Unchanged	82 (40)	39 (51)	
Better	71 (35)	14 (18)	0.040

^a^Linear-by-Linear Association

^b^3 missing in the intervention group and 5 missing in the referent group

^c^6 missing in the intervention group and 1 missing in the referent group

Values presented are number (%)

When the data from the questionnaire group were dichotomized into two groups: those with low or high frequency of neck/shoulder complaints at baseline, the results showed a significant improvement in perceived exertion for those in the intervention group with a high frequency of complaints at baseline who used the prismatic glasses on a regular basis (*p* = 0.040), compared to those in the reference group reporting the same frequency of complaints at baseline (Table 3). Moreover, the results for the high compliers with low frequency of complains at baseline also indicated a trend (although not statistically significant) towards a preventive effect of the prismatic glasses, since this group showed less perceived exertion over time compared to the reference group (Table 3).

**Table 3:** Changes between baseline and follow-up among participants in the questionnaire survey, in frequency of neck/shoulder complaints and perceived exertion. Data is shown for the Intervention group^a^ and the Reference group, stratified by frequency of complaints at baseline

	Frequency of complaints at baseline					
	Twice a month or less			Once a week or more		
	Intervention group	Reference group	p-value^b^	Intervention group	Reference group	p-value^b^
Questionnaire	*n* = 60	*n* = 58		*n* = 110	*n* = 70	
Frequency of complaints						
Higher	7 (12)	18 (31)		5 (5)	8 (11)	
Unchanged	42 (70)	29 (50)		49 (45)	28 (40)	
Lower	11 (18)	11 (19)	0.112	56 (51)	34 (49)	0.392
Perceived exertion^c,d^						
Worse	19 (33)	22 (41)		19 (17)	22 (32)	
Unchanged	10 (17)	17 (31)		27 (25)	15 (22)	
Better	29 (50)	15 (28)	0.083	64 (58)	32 (46)	0.040

^a^66 participants who were given prismatic glasses but reported that they did not use them on a regular basis, were excluded

^b^Linear-by-Linear Association

^c^Twice a month or less; 2 subjects missing in the intervention group and 4 missing in the reference group

^d^Once a week or more; 1 subject missing in the reference group

Values presented are number (%)

When dichotomizing according to neck pain at baseline in those who underwent the HECO examinations, the results from the pain group showed a strong trend towards improvements regarding clinical diagnoses (*p* = 0.006), and a significant improvement in work ability (*p* = 0.002) in the intervention group, compared to the reference group (Table 4).

**Table 4:** Changes between baseline and follow-up among the participants in the clinical examination, in neck/shoulder pain, diagnosed disorders and work ability. Data is shown for the Intervention group^a^ and the Reference group, stratified by neck/shoulder pain at baseline (no/yes)

	Neck/shoulder pain at baseline					
	No			Yes		
	Intervention group	Reference group	p-value^b^	Intervention group	Reference group	p-value^b^
Clinical examination (HECO)	*n* = 85	*n* = 52		*n* = 102	*n* = 36	
Musculoskeletal pain^c^						
Worse	15 (18)	13 (25)				
Unchanged	68 (82)	39 (75)	0.386	67 (68)	27 (75)	
Better				32 (32)	9 (25)	0.527
One or more diagnoses						
Worse	7 (8)	3 (6)		7 (7)	6 (17)	
Unchanged	78 (92)	49 (94)	0.742	74 (73)	29 (81)	
Better				21 (21)	1 (3)	0.006
Work ability^d^						
Worse	16 (22)	8 (20)		23 (25)	15 (42)	
Unchanged	38 (51)	22 (55)		32 (34)	17 (47)	
Better	20 (27)	10 (25)	1.000	38 (41)	4 (11)	0.003

^a^45 participants who were given prismatic glasses but reported that they did not use them on a regular basis were excluded

^b^Linear-by-Linear Association

^c^Five subjects missing in the intervention group; whereof two without pain and three with pain at baseline

^d^Twenty subjects missing in the Intervention group (11 without pain and 9 with pain at baseline). Twelve subjects from the Reference group missing, all without pain

Values presented are number (%)

Compliance in the use of the prismatic glasses was fairly high over time. Approximately 75 % reported that they used them on a regular basis (every day). Spontaneous comments from the participants in the intervention group indicated that the greatest advantage of the prismatic glasses was during root-fillings and other vision-demanding tasks in constrained postures.

## Discussion

### Principal findings

By using prismatic glasses in dental work it seem to be possible, to reduce self-reported complaints, as well as clinically defined diagnoses in the neck/shoulders among dental professionals. Moreover, it is, according to our results, possible to decrease self-reported perceived exertion during clinical dental work, which was most prominent among those who reported neck pain at baseline. Furthermore, our results indicated less aggravation of neck/shoulder symptoms in the intervention group than in the reference group. Lastly, a significant increase in self-reported work ability was seen among participants in the intervention group compared to the reference group 12 months after the implementation of prismatic glasses in everyday clinical practice.

### The results in relation to other studies

To the best of our knowledge, few other ergonomic interventions have led to similar results with respect to long-term effects on neck pain. According to the most recent review of the effectiveness of physical ergonomic interventions on neck pain, no differences are usually found in the prevalence/incidence of neck pain between workers who were the subject of an ergonomic intervention and those who were not [46]. Furthermore, this applied to both the short-term (6 months) and in long-term (12 months) perspective. However, there is a lack of high-quality studies, partly due to the well-known difficulties associated with large-scale intervention studies “in real life”, and thus outside the absolute control of the research team. However, it is of the utmost importance to resolve these problems if we are to elucidate and validate the effects of ergonomic interventions in the “natural” context.

The intervention in the present study was a classical ergonomic intervention, where an available and previously evaluated intervention (prismatic glasses) was used to reduce a well-known risk factor among dental professionals. The positive effects of prismatic glasses on exposure regarding forward bending of the neck had already been confirmed in a randomised controlled study performed on a subgroup from the same study population [34]. In contrast to these positive results a recently published study on loupes, which until now have been the most conventional way to solve ergonomic problems, has concluded that wearing loupes have both positive and negative effects on neck pain [32]. In our experience, the way we performed this study, in two steps at first the RCT-study on exposure than a population based study on effects of the intervention is a constructive approach to convincing employers of the benefits of investing in ergonomic interventions, in the current economic climate. Similarly, a systematic review of the effectiveness of interventions involving the use of suitable chairs in the workplace to reduce musculoskeletal symptoms had identified the necessity of reliable evidence of the benefits that can be expected from the investments associated with such interventions [47]. Another systematic review has previously pointed out that many intervention studies are of low quality since they lack information on compliance or do not report those data [46]. In our study, we were able to follow the self-reported compliance with the prismatic glasses 12 month after the intervention. This enabled us to confirm that the long-term effects of the intervention on neck pain and neck diagnoses were even better when only participants with good compliance were included in the data analysis. The high compliance reported by the intervention group, together with spontaneous comments regarding the facilitative effects of the glasses in everyday work, indicated that the beneficial effects on neck pain, perceived exertion and visual comfort outweighed initial problems of adjustment such as optical phenomena, poor fitting, feeling of dizziness, etc.

The significant decrease in self-reported exertion among participants in the intervention group who reported neck pain at baseline, and the tendency towards the same decrease (although not statistically significant) among those who reported more occasional episodes of neck pain (≤2 times a month) indicated both a primary preventive effect as well as secondary effect of the prismatic glasses on neck pain. Previous research has identified the importance of reducing perceived exertion since high perceived exertion during work has been identified in earlier studies as a predictor of future pain and long-lasting pain/symptoms in the musculoskeletal system, both in professions with similar exposure to vision-demanding work tasks (computer users) [36], and in those exposed to more “traditional” physical loads (heavy lifting among assistant nurses) [48].

Finally, our results showed a significantly increased self-reported work ability in the intervention group compared to the reference group. It is likely that the decrease in neck pain in the intervention group was a major contributor to the increased work ability. Since there may be other unknown factors influencing the participants’ perception of work ability, this result should be interpreted with caution. However, several studies have explored the relation between work ability, and musculoskeletal pain either in combination with, for instance, stress or pain alone. The conclusion of this study is that musculoskeletal pain in itself is negatively related to perceived work ability [49, 50].

### Strengths and weaknesses of the study

The major strength of this study is the longitudinal design. Another strength is the relatively long follow-up period (12 months), which allowed us to explore the long-term effects of the intervention. Also the use of two different assessment methods could be considered as a strength.

An obvious limitation of this study is the lack of randomization between the two groups. Such an approach was not possible due to organizational and financial reasons. However, from a practical point of view, studies performed in a clinical setting are much needed because they have a high degree of external validity and provide findings that are more generalizable than those obtained from randomized controlled studies. Not surprisingly, subjects with ongoing pain volunteered to a higher degree than others to try the prismatic spectacles. When stratifying by pain status at baseline, we found a clear reduction in symptoms resulting from the use of prismatic glasses among those with ongoing pain, and a tendency towards reduced perceived exertion among those without pain who used the glasses on a regular basis. Furthermore, since the baseline measurements showed no major differences between the intervention group and the reference group concerning other relevant background factors, we consider the results reliable.

Another limitation could be the dichotomised handling of the data which might have reduced the amount of available information, and thus affected the sensitivity of the outcome measures. However, we made the evaluation that in this clinical context this approach would be reasonable to use.

In order to avoid possible examiner bias concerning the HECO examination before and after the intervention, the participants were told not to reveal to the ergonomists which group they belonged to at any time. However, despite this precaution, we are aware of the possibility of potential bias. Finally, the use of two different methods, an objective method (clinical examinations) and a subjective method (self-reported data) with respect to our outcomes is, in our opinion, a strength since the two methods point in the same direction.

### Possible mechanisms and practical implications

We believe that the mechanism responsible for these findings, is largely the reduction in exposure to extreme forward bending of the neck resulting from the use of prismatic spectacles. Furthermore, a more relaxed working technique due to better visual ergonomics might explain part of the decline in neck pain. However, it could not be completely ruled out that the effects also could relate to unspecific factors involving different aspects of being paid attention to (Hawthorne effect). But, since our results are to a great extend built on clinical examinations and not only self-reports this potential bias was in our opinion minimized.

## Conclusion

Thus, the conclusion of this study is that dental personnel opting to wear prismatic spectacles reduced their neck pain significantly more at follow up compared with the reference group. Furthermore, it is worthwhile testing in a randomised design in order to be able to confirm this conclusion. The practical implication of this study is that recommendations regarding ergonomics for dental professionals should include the use of prismatic glasses, both as primary and secondary prevention of work-related neck pain. Such glasses should also be tested in other working situations where the work tasks include high visual demands in sustained awkward neck postures.

## Additional file

Additional file 1: Photo of prismatic glasses. (DOCX 118 kb)

## References

[1] Andersen JH, Kaergaard A, Mikkelsen S, Jensen UF, Frost P, Bonde JP, et al. Risk factors in the onset of neck/shoulder pain in a prospective study of workers in industrial and service companies. Occup Environ Med. 2003;60(9):649–54. 10.1136/oem.60.9.649 PMC1740607 12937185

[2] Kinge JM, Knudsen AK, Skirbekk V, Vollset SE (2015). Musculoskeletal disorders in Norway: prevalence of chronicity and use of primary and specialist health care services. BMC Musculoskelet Disord.

[3] Pernold G, Tornqvist EW, Wiktorin C, Mortimer M, Karlsson E, Kilbom A, et al. Validity of occupational energy expenditure assessed by interview. AIHA J (Fairfax, Va). 2002;63(1):29–33. 10.1080/15428110208984688 11843422

[4] Alavinia SM, van Duivenbooden C, Burdorf A (2007). Influence of work-related factors and individual characteristics on work ability among Dutch construction workers. Scand J Work Environ Health.

[5] Alexopoulos ECSI, Charizani F (2004). Prevalence of musculoskeletal disorders in dentists. BMC Musculoskelet Disord.

[6] Nordander C, Ohlsson K, Akesson I, Arvidsson I, Balogh I, Hansson GA, et al. Risk of musculoskeletal disorders among females and males in repetitive/constrained work. Ergonomics. 2009;52(10):1226–39. 10.1080/00140130903056071 19787502

[7] Wigaeus Hjelm E, Karlqvist L, Hagberg M, Hagman M, Hansson Risberg E, Isaksson A, et al. Working conditions and musculoskeletal disorders among male and female computer operators. In: XIV Triennal Congress of the International Ergonomics Society and 44th Annual Meeting of the Human Factors and Ergonomics Society: July 29th-August 4th 2000. San Diego: Human Factors and Ergonomics Society; 2000. p. 675–7.

[8] Luime JJ, Kuiper JI, Koes BW, Verhaar JAN, Miedema HS, Burdorf A. Work-related risk factors for the incidence and recurrence of shoulder and neck complaints among nursing-home and elderly-care workers. Scand J Work Environ Health. 2004;30(4):279–86. 10.5271/sjweh.795 15458010

[9] Morse T, Bruneau H, Dussetschleger J (2010). Musculoskeletal disorders of the neck and shoulder in the dental professions. Work.

[10] Ariens GA, van Mechelen W, Bongers PM, Bouter LM, van der Wal G (2001). Psychosocial risk factors for neck pain: a systematic review. Am J Ind Med.

[11] Bernal D, Campos-Serna J, Tobias A, Vargas-Prada S, Benavides FG, Serra C (2015). Work-related psychosocial risk factors and musculoskeletal disorders in hospital nurses and nursing aides: a systematic review and meta-analysis. Int J Nurs Stud.

[12] Bongers PM, Ijmker S, van den Heuvel S, Blatter BM (2006). Epidemiology of work related neck and upper limb problems: psychosocial and personal risk factors (part I) and effective interventions from a bio behavioural perspective (part II). J Occup Rehabil.

[13] Arvidsson I, Axmon A, Skerfving S (2008). Follow-up study of musculoskeletal disorders 20 months after the introduction of a mouse-based computer system. Scand J Work Environ Health.

[14] Gustafsson E, Johnson PW, Lindegård A, Hagberg M.Technique, muscle activity and kinematic differences in young adults texting on mobile phones. Ergonomics 2011;54(5):477-87. 10.1080/00140139.2011.568634 21547792

[15] Harrington CB, Siddiqui A, Feuerstein M (2009). Workstyle as a predictor of pain and restricted work associated with upper extremity disorders: a prospective study. J Hand Surg [Am].

[16] Huysmans MA, Blatter BM, van der Beek AJ (2012). Perceived muscular tension predicts future neck-shoulder and arm-wrist-hand symptoms. Occup Environ Med.

[17] Åkesson I, Balogh I, Hansson GA. Physical workload in neck, shoulders and wrists/hands in dental hygienists during a work-day. Appl Ergon. 2012;43(4):803–11. 10.1016/j.apergo.2011.12.001 22208356

[18] Hayes M, Cockrell D, Smith DR (2009). A systematic review of musculoskeletal disorders among dental professionals. Int J Dent Hyg.

[19] Jadhav SKJR, Dhumal PS, Tillu G, Hegde VS (2015). Prevalence and prevention of musculoskeletal pain in conservative dentistry and endodontics: An online survey. Univ Res J Dent.

[20] Jonker D, Rolander B, Balogh I (2009). Relation between perceived and measured workload obtained by long-term inclinometry among dentists. Appl Ergon.

[21] Ariens GA, Bongers PM, Douwes M, Miedema MC, Hoogendoorn WE, van der Wal G, et al. Are neck flexion, neck rotation, and sitting at work risk factors for neck pain? Results of a prospective cohort study. Occup Environ Med. 2001;58(3):200–7. 10.1136/oem.58.3.200 PMC1740110 11171934

[22] Lorusso A, Bruno S, L’Abbate N (2009). [Musculoskeletal disorders among university student computer users]. Med Lav.

[23] Gupta S (2011). Ergonomic applications to dental practice. Indian J Dent Res.

[24] Hayes MJ, Osmotherly PG, Taylor JA, Smith DR, Ho A (2014). The effect of wearing loupes on upper extremity musculoskeletal disorders among dental hygienists. Int J Dent Hyg.

[25] Rafeemanesh E, Jafari Z, Kashani FO, Rahimpour F (2013). A study on job postures and musculoskeletal illnesses in dentists. Int J Occup Med Environ Health.

[26] Rafie F, Zamani Jam A, Shahravan A, Raoof M, Eskandarizadeh A (2015). Prevalence of upper extremity musculoskeletal disorders in dentists: symptoms and risk factors. J Environ Public Health.

[27] Åkesson I, Hansson GA, Balogh I, Moritz U, Skerfving S. Quantifying work load in neck, shoulders and wrists in female dentists. Int Arch Occup Environ Health. 1997;69(6):461-74 . 10.1007/s004200050175 9215934

[28] Aaras A, Horgen G, Ro O, Loken E, Mathiasen G, Bjorset HH, et al. The effect of an ergonomic intervention on musculoskeletal, psychosocial and visual strain of VDT data entry work: the Norwegian part of the international study. Int J Occup Saf Ergon. 2005;11(1):25–47. 10.1080/10803548.2005.11076627 15794872

[29] Hemphala H, Nylen P, Eklund J (2014). Optimal correction in spectacles: intervention effects on eyestrain and musculoskeletal discomfort among postal workers. Work.

[30] Hemphälä H, Eklund J (2011). A visual ergonomics intervention in mail sorting facilities: effects on eyes, muscles and productivity. Appl Ergon.

[31] Horgen G, Aaras A, Thoresen M (2004). Will visual discomfort among visual display unit (VDU) users change in development when moving from single vision lenses to specially designed VDU progressive lenses?. Optom Vis Sci.

[32] Hayes MJ, Osmotherly PG, Taylor JA, Smith DR, Ho A (2016). The effect of loupes on neck painand disability among dental hygienists. Work.

[33] Hayes M, Taylor J, Smith D. Introducing loupes to clinical practice: dental hygienists experiences and opinions. Int J Dent Hyg. 2016;14(3):226-30. 10.1111/idh.12128 25690424

[34] Lindegård A, Gustafsson M, Hansson GA (2012). Effects of prismatic glasses including optometric correction on head and neck kinematics, perceived exertion and comfort during dental work in the oral cavity--a randomised controlled intervention. Appl Ergon.

[35] Stock SR, Fernandes R, Delisle A, Vezina N (2005). Reproducibility and validity of workers’ self-reports of physical work demands. Scand J Work Environ Health.

[36] Lindegård A, Wahlström J, Hagberg M, Vilhelmsson R, Toomingas A, Wigaeus Tornqvist E (2012). Perceived exertion, comfort and working technique in professional computer users and associations with the incidence of neck and upper extremity symptoms. BMC Musculoskelet Disord.

[37] Jonker D, Gustafsson E, Rolander B, Arvidsson I, Nordander C (2015). Health surveillance under adverse ergonomics conditions - validity of a screening method adapted for the occupational health service. Ergonomics.

[38] Kuorinka I, Jonsson B, Kilbom A, Vinterberg H, Biering-Sorensen F, Andersson G, et al. Standardised Nordic questionnaires for the analysis of musculoskeletal symptoms. Appl Ergon. 1987;18(3):233–7. 10.1016/0003-6870(87)90010-x 15676628

[39] Holmstrom E, Moritz U (1991). Low back pain--correspondence between questionnaire, interview and clinical examination. Scand J Rehabil Med.

[40] Borg G (1990). Psychophysical scaling with applications in physical work and the perception of exertion. Scand J Work Environ Health.

[41] Arvidsson I, Simonsen JG, Dahlqvist C, Axmon A, Karlson B, Björk J, et al. Cross-sectional associations between occupational factors and musculoskeletal pain in female teachers, nurses and sonographers. BMC Musculoskelet Disord. 2016;17:35. 10.1186/s12891-016-0883-4 PMC4717636 26781760

[42] Tuomi K, Ilmarinen J, Martikainen R, Aalto L, Klockars M (1997). Aging, work, life-style and work ability among Finnish municipal workers in 1981–1992. Scand J Work Environ Health.

[43] Johansson G, Hultin H, Möller J, Hallqvist J, Kjellberg K. The impact of adjustment latitude on self-assessed work ability in regard to gender and occupational type. Scand J Occup Ther. 2012;19(4):350-9. 10.3109/11038128.2011.603354 21854104

[44] Slebus FG, Kuijer PP, Willems JH, Sluiter JK, Frings-Dresen MH (2007). Prognostic factors for work ability in sicklisted employees with chronic diseases. Occup Environ Med.

[45] Åhlström L, Grimby-Ekman A, Hagberg M, Dellve L (2010). The work ability index and single-item question: associations with sick leave, symptoms, and health--a prospective study of women on long-term sick leave. Scand J Work Environ Health.

[46] Driessen MT, Proper KI, van Tulder MW, Anema JR, Bongers PM, van der Beek AJ (2010). The effectiveness of physical and organisational ergonomic interventions on low back pain and neck pain: a systematic review. Occup Environ Med.

[47] van Niekerk SM, Louw QA, Hillier S (2012). The effectiveness of a chair intervention in the workplace to reduce musculoskeletal symptoms. A systematic review. BMC Musculoskelet Disord.

[48] Andersen LL, Clausen T, Persson R, Holtermann A (2012). Perceived physical exertion during healthcare work and prognosis for recovery from long-term pain in different body regions: Prospective cohort study. BMC Musculoskelet Disord.

[49] Lindegard A, Larsman P, Hadzibajramovic E, Ahlborg G (2014). The influence of perceived stress and musculoskeletal pain on work performance and work ability in Swedish health care workers. Int Arch Occup Environ Health.

[50] Oberlinner C, Yong M, Nasterlack M, Pluto RP, Lang S (2015). Combined effect of back pain and stress on work ability. Occup Med (Lond).

