# Supplementary figures and images for: Opting to wear prismatic spectacles was associated with reduced neck pain in dental personnel: a longitudinal cohort study
Source: BMC Musculoskelet Disord. 2016 Aug 17;17:347. doi: 10.1186/s12891-016-1145-1 (PMC4989289; doi:10.1186/s12891-016-1145-1)

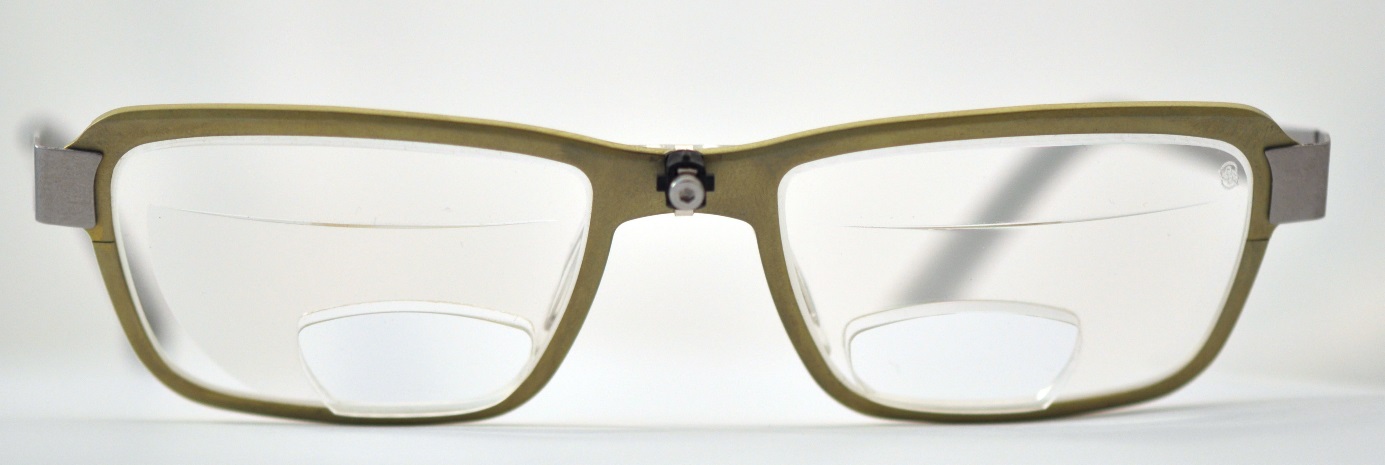

Supplement: Additional file 1: — Photo of prismatic glasses. (DOCX 118 kb) [file 12891_2016_1145_MOESM1_ESM.docx]
